# Determination of *NAT2* Genotypes in a Cohort of Patients with Suspected TB in the State of Rio de Janeiro

**DOI:** 10.3390/pharmaceutics16070917

**Published:** 2024-07-10

**Authors:** Cecília Alvim Dutra, Raquel Lima de Figueiredo Teixeira, Márcia Quinhones Pires Lopes, Victória de Moraes Silva, Philip Noel Suffys, Ricardo de Souza Carvalho, Adriana Rezende Moreira, Adalberto Rezende Santos, Afrânio Lineu Kritski

**Affiliations:** 1 Academic Tuberculosis Program, Faculty of Medicine, HUCFF-IDT Hospital Complex, Federal University of Rio de Janeiro, Rio de Janeiro 21941-913, Brazil; cecialvim93@gmail.com (C.A.D.); rezendemoreira.adriana@gmail.com (A.R.M.); kritskia@gmail.com (A.L.K.); 2 Laboratory of Molecular Biology Applied to Mycobacteria, Oswaldo Cruz Institute, IOC/Fiocruz, Avenida Brasil, 4365, Rio de Janeiro 21040-360, Brazil; ft.raquel@gmail.com (R.L.d.F.T.); mqplopes@gmail.com (M.Q.P.L.); victoriamoraess97@gmail.com (V.d.M.S.); psuffys@gmail.com (P.N.S.); 3 Department of General Medicine, University of Rio de Janeiro (UniRio), Rio de Janeiro 20270-330, Brazil; ricardo.carvalho@unirio.br

**Keywords:** tuberculosis, single-nucleotide polymorphisms, pharmacogenetics, treatment, *NAT2*

## Abstract

The human N-acetyltransferase 2 enzyme, encoded by the *NAT2* gene, plays an important role in the metabolism of isoniazid, the main drug used to treat tuberculosis. The interindividual variation in the response of patients to drug treatment for tuberculosis may be responsible for the occurrence of unfavorable outcomes. The presence of polymorphisms in genes associated with the metabolism and transport of drugs, receptors, and therapeutic targets has been identified as a major determinant of this variability. The objective of this study was to identify the genetic profile of *NAT2* in the study population. Using the obtained genomic DNA followed by PCR amplification and sequencing, the frequency of nine SNPs as well as alleles associated with slow (47.9%), intermediate (38.7%), and fast acetylation phenotypes (11.3%), in addition to those whose phenotype has not yet been characterized (2.1%), was estimated. The NAT2*5B allele was identified more frequently (31.3%). The description of SNPs in pharmacogenes and the establishment of their relationship with the pharmacokinetics of an individual offer an individualized approach that allows us to reduce the unfavorable outcomes of a therapy, ensure better adherence to treatment, prevent the emergence of MDR strains, reduce the cost of treatment, and improve the quality of patients’ lives.

## 1. Introduction

Tuberculosis is an infectious disease caused by *Mycobacterium tuberculosis,* and it ranks as the second leading cause of death globally, just behind COVID-19. In 2021, it was estimated that around 10.6 million people worldwide were diagnosed with tuberculosis, reflecting a 4.5% increase compared to the 10.1 million cases reported in 2020.

The incidence rate of tuberculosis also showed a 3.6% increase from 2020 to 2021, reversing a declining trend that had been observed over the previous two decades, with an average annual reduction of approximately 2% in tuberculosis cases. Approximately 71% of individuals (2.4 out of every 3.4 million) diagnosed through bacteriological confirmation underwent rifampicin resistance testing, maintaining the same coverage level as in 2020 (2.1 out of every 3 million) and showing an increase compared to 2019 (61%, or 2.2 out of every 3.6 million). In total, 141,953 cases of multidrug/rifampicin-resistant tuberculosis (TB-MDR/TB-RR) and 25,038 cases of extensively drug-resistant tuberculosis (TB-XDR) were identified, amounting to a total of 166,991 cases. This represented a 6.4% increase compared to the 2020 total of 156,982, but still represents a 17% decrease compared to the 2019 total of 201,997 [1].

The COVID-19 pandemic posed a significant challenge to the global efforts toward combating tuberculosis, which aimed to reduce the number of deaths from the disease over the years. The limited access to tuberculosis diagnosis and treatment during the pandemic resulted in an increase in the number of deaths despite a decrease in reported cases. The number of individuals receiving treatment for multidrug-resistant tuberculosis (TB-MDR) showed a 17% decline, decreasing from 181,533 to 150,469 (corresponding to approximately one in three people in need of treatment), with a partial recovery of 7.5%, corresponding to 161,746, in 2021 [1].

The necessary COVID-19 pandemic restrictive measures, such as lockdowns, and the behavioral changes adopted, such as mask wearing, may have contributed to the reduction in tuberculosis transmission in 2020 and 2021. However, several other factors played a crucial role in reducing the number of reported cases, including the limited capacity of the healthcare system to maintain the provision of follow-up services (reduced in-person visits and reliance on remote support, as well as the redirection of resources to combat the COVID-19 pandemic), the population’s reduced willingness to seek care during lockdowns (amplified by concerns about the risks of visiting healthcare facilities during a pandemic), and the stigma associated with the similarities in symptoms between tuberculosis and COVID-19 [1].

In 2021, Brazil recorded 68,271 new tuberculosis cases, with an incidence rate of 32 cases per 100,000 inhabitants and a cure rate of 65.4% [2]. Brazil is considered a priority country in tuberculosis control, partly due to its membership in the BRICS group, an economic consortium of emerging countries consisting of Brazil, Russia, India, China, and South Africa, representing approximately 50% of tuberculosis cases worldwide. Additionally, Brazil is among the 30 countries with the highest burden of tuberculosis and tuberculosis–HIV coinfection [3].

According to the Epidemiological Bulletin of the Ministry of Health, in 2021, significant heterogeneity in the incidence of tuberculosis was observed, with 11 states reporting rates higher than the national average. Rio de Janeiro has the second-highest rate of tuberculosis cases in the country, having recorded 15,937 cases, including 12,796 new cases [2,4].

Although tuberculosis is a treatable disease, with therapeutic success rates of approximately 85% upon completing treatment, there are often less-favorable outcomes, including adverse reactions, delays, or treatment failure, which can be attributed to variations in drug bioavailability, such as isoniazid and rifampicin, for different patients. [5,6]. It is known that the differences in individual responses to drug treatment can be attributed to single-nucleotide polymorphisms (SNPs) found in genes related to drug metabolism, transport, receptors, and therapeutic targets [7].

The *NAT2* gene encodes the enzyme N-acetyltransferase 2, a phase II biotransformation enzyme predominantly expressed in the liver, small intestine, and colon. NAT2 is a 33.79 kDa cytosolic enzyme responsible for the N-acetylation and O-acetylation of arylamine, hydrazine, and heterocyclic amines. This process occurs through the transfer of the acetyl group from the acetyl-Coenzyme A (acetyl-CoA) cofactor to the nitrogen terminal of xenobiotics. NAT2 plays a crucial role in the metabolism and inactivation of carcinogens and drugs used in the treatment of infections and chronic diseases, including tuberculosis, leprosy, and arterial hypertension, among others [8,9]. Located at position 22 on the short arm of chromosome 8, the *NAT2* gene contains 873 base pairs (bp) in its coding region and is highly polymorphic. NAT2 alleles are determined by the combination of one to six SNPs within the same gene locus. These alleles are then grouped into clusters, each having a specific SNP in common (while others vary). The genotypes are defined by pairs of alleles that may or may not belong to the same cluster [10].

The different *NAT2* genotypes enable us to categorize individuals based on their metabolic profiles into three groups: (i) fast acetylators, who may exhibit an elevated production of the metabolizing enzyme, leading to insufficient drug disposition in the body, which can make it challenging to effectively treat the illness; (ii) intermediate acetylators, who possess balanced drug metabolism, resulting in successful therapeutic outcomes; and (iii) slow acetylators, characterized by decreased or absent levels of the metabolizing enzyme. They may experience adverse reactions due to the accumulation of the drug and/or its metabolites [11].

The influence of SNPs within the *NAT2* gene on the pharmacokinetics of isoniazid is well established, particularly concerning slow acetylators and their susceptibility to drug-induced hepatitis [12]. Conversely, fast acetylators, who metabolize isoniazid more quickly, have been associated with lower drug concentrations in the blood. This can potentially lead to treatment delays and failures, as the therapeutic effect of isoniazid is linked to achieving sufficient drug concentrations [13]. Research teams from various countries have examined this relationship and suggested personalized therapies based on a given patient’s genotype [13,14,15,16,17].

Given the significant variability of the *NAT2* gene in different populations, as recently described in [18], and the association of SNPs with various treatment outcomes for drugs metabolized by N-acetyltransferase 2, the goals of this study were to partially map the coding region of the *NAT2* gene in a Rio de Janeiro cohort and identify SNPs that could potentially serve as biomarkers for predicting therapeutic responses and proposing revised dosages of INH. We believe that identifying such markers could contribute to optimizing tuberculosis treatment, enhancing its safety and efficacy [18].

## 2. Materials and Methods

This descriptive cohort study was conducted through collaboration between the Municipal Department of Duque de Caxias and the Academic Tuberculosis Program (PAT) of the Medical School (FM) and the Institute of Thorax Diseases (IDT) hospital complex and Clementino Fraga Filho Hospital (HUCFF) of the Federal University of Rio de Janeiro (UFRJ).

The samples included in the study were retrospectively selected based on data extracted from the database of the Municipal Health Center of Duque de Caxias (RJ) for suspected pulmonary TB cases spanning from 2016 to 2020. A total of 304 individuals being treated at the center during that period were included in this study. Among them, TB was clinically and laboratory-confirmed (through bacilloscopy, RX images, and culture) for 136 patients (44.7%), who subsequently underwent conventional first-line treatment, with no interruptions. Demographic, clinical, and laboratory data were collected and managed using the REDCap (Research Electronic Data Capture) platform. As unfavorable outcomes, we considered positive BAAR/culture and worsening/no improvement in radiographic images after 2 months of treatment.

This study was approved by the UFRJ’s ethics committee. Patients were selected based on the following inclusion criteria: (a) being over 18 years of age; (b) having been diagnosed with active pulmonary tuberculosis with positive sputum culture (with or without positive AFB smear or Xpert^®^ MTB/RIF assessment); and (c) having a new or recurrent case of TB.

### 2.1. DNA Extraction and Genotyping

Peripheral blood samples were collected from 304 individuals suspected of having TB and stored at −20 °C. Subsequently, genomic DNA was obtained from 200 µL of the stored blood using the “QIAamp DNA blood^®^” commercial kit produced by QIAGEN (Germantown, MD, USA), following the manufacturer’s instructions.

The primers used for amplification and sequencing of the *NAT2* gene were designed using the Primer3Plus program based on reference sequences obtained from the National Center for Biotechnology Information (NCBI) database (RefSeq: NCBI Reference Sequence Database) [9]. The fragment of interest encompassing the coding region of the *NAT2* gene was amplified via PCR (1083 bp) using two external primers: 5’ TTAGTCACACGAGGAAATCAAA 3’ (forward) and 5’ AAATGCTGACATTTTTATGGATGA 3’ (reverse).

The PCR amplification was achieved in 50 µL of a reaction mixture containing 50 ng of genomic DNA, 1X Rxn buffer (Invitrogen™, Waltham, MA, USA), 1.5 mM of MgCl_2_, 0.2 mM of dNTPs, 200 ng of each primer, 1 U of Taq polymerase platinum (Invitrogen™), and deionized water. Samples were incubated at 94 °C for 5 min, followed by 20 cycles of 94 °C for 1 min; 67 °C for 1 min; and 72 °C for 1 min. Subsequently, there were 15 cycles of 94 °C for 1 min; 57 °C for 1 min; and 72 °C for 1 min. The final extension was conducted at 72 °C and lasted 5 min. The purification of the obtained products was carried out using the “Charge Switch^®^ PCR Clean-Up” kit (Invitrogen). These products were then visualized after electrophoresis in 1.5% agarose gel to verify the integrity and correct size of the amplicons.

Automated sequencing reactions were performed using the “ABI PRISM^®^ Big Dye^®^ Terminator v3.1 kit”, which was used according to the manufacturer’s instructions. A total of four reactions were prepared for each sample, using both the external amplification primers and internal primers: 5′ACCATTGACGGCAGGAATTA 3′ (forward) and 5′ TGGTCCAGGTACCAGATTC 3′ (reverse) [9]. Subsequently, the sequences were read using a 48-capillary sequencer “ABI PRISM^®^ 3730 Genetic Analyzer” (Applied Biosystems, Waltham, MA, USA). The obtained results were then analyzed using SeqScape software, version 2.5, developed by Applied Biosystems.

### 2.2. Statistical Analysis

Haplotype reconstruction, genotype identification, and inferencing of NAT2 phenotypes were conducted using the PHASE v2.1.1 software product [19], which employs a Bayesian statistical approach to calculate the combinations of alleles present on each of the two chromosomes in diploid individuals.

The primary outcomes considered are the occurrences throughout the treatment, that is, unfavorable outcomes such as positive culture/bacilloscopy results remaining at the second month and delayed radiographic improvement (TB group). These outcomes were associated with each identified SNP in the *NAT2* gene. Logistic regression control was performed for potential confounding variables, namely, *Mycobacterium tuberculosis* strain (MTB lineage) and disease severity assessed via chest radiography (extent of the image in thirds as well as bilateral or cavitary disease at the beginning and 2 months after the start of treatment).

After describing the alleles and haplotypes of the *NAT2* gene and defining the phenotypic inference of the acetylation profile in the study population, an analysis of the association between the identified SNPs and the occurrence of complications during TB treatment was conducted using the chi-square test and odds ratio calculation. A comparison between the presence of SNPs and the treatment duration (variables with a non-normal distribution) was performed using the Mann–Whitney U test.

All analyses were conducted using the IBM SPSS Statistics 23.0 software, applying a significance level of 0.05 between expected and observed values.

## 3. Results

### 3.1. SNP Description

Nine SNPs were identified within the *NAT2* gene (Table 1), with seven of them being commonly observed in diverse populations, namely, c.191G>A, c.341T>C, c.590G>A, and c.857G>A, which lead to a reduced acetylation capacity due to amino acid changes, and c.282C>T, c.481C>T, and c.803A>G, wherein no amino acid exchange occurs, and the enzyme’s function remains unaltered [11,20].

### 3.2. Haplotype Reconstruction and Characterization of NAT2 Gene Alleles

The SNPs identified in the *NAT2* gene were analyzed using the Phase software v.2.1.1 product, resulting in the characterization of 19 alleles (Table 2) associated with different acetylation phenotypes, including fast (35.7%) and slow (64.3%) phenotypes.

The most frequent alleles in both the TB and non-TB cases were NAT2*5B (31.3%), NAT2*6A (23.1%), and NAT2*4 (22.3%). The alleles NAT2*new5 (0.5%) and NAT2*new6 (1%) were given these names because they share the SNP common to the cluster *5 (c.341T>C) and *6 (c.590G>A), respectively. However, the combination of these SNPs with others identified in the same sequence has not been characterized in previous studies; thus, their impact on the acetylation phenotype remains unknown.

The next phase of haplotypic reconstruction involves statistically combining the likely alleles within each sample to infer genotypes. In this population, a total of 46 unique genotypes were identified, categorizing individuals into three distinct acetylation phenotypes: fast (13%), intermediate (34.8%), and slow (39.2%). Some allele combinations result in an undetermined phenotype (13%), as the specific impact and alterations caused by the combined SNPs in these alleles are not known (Table 3).

### 3.3. Association between SNPs and Unfavorable Outcomes in the Second Month of Treatment among Patients with Pulmonary TB and the Duration of Anti-TB Treatment

An analysis of the association between allelic variants (in terms of heterozygosity or homozygosity) in the *NAT2* gene and the occurrence of complications during TB treatment was carried out using the chi-square test and odds ratio calculation. Based on the significance level considered (*p* < 0.05), no statistically significant differences were observed (Table 4).

The comparison between the presence of SNPs and the treatment duration was conducted using the Mann–Whitney U test, a non-parametric statistical test for comparing independent groups. As shown in Table 5, the association of the number of months re-quired for treatment effectiveness among patients with a mutant allele was observed only in the case of the *NAT2* c.857G>A SNP. This SNP was more frequent in the group with a treatment duration of less than 9 months, implying its potential role as a genetic marker indicative of the effectiveness of anti-TB treatment within a six-month period.

## 4. Discussion

Nine variants in the *NAT2* gene were identified, with c.341T>C and c.282C>T being the SNPs observed in higher frequencies (59.9% and 56.2%, respectively) among the individuals included in this descriptive study. The observed frequencies align with data from the Brazilian literature, with one exception, namely, the c.191G>A variant, which was found less frequently (2.3%). This deviation may be attributed to our limited sample size. This particular variant characterizes the NAT2*14 cluster associated with a slow acetylation phenotype, which is more prevalent in the African continent [11,21,22]. In this study, the representative of this cluster, the NAT2*14B allele (c.191G>A rs1801279 + c.282C>T rs1041983), was identified at a frequency of 2.4%.

The frequency of SNPs in the *NAT2* gene exhibits variability among different populations, posing challenges for establishing a standardized dose of isoniazid. For instance, the East Asian population (including China, Korea, and Japan) is relatively more homogeneous, displaying fewer mutations. Within this population, the c.857G>A variant and the NAT*4 allele are highly prevalent, leading to a predominance of the intermediate acetylation phenotype (46%). This is a consequence of the genotype, which combines a fast allele and a slow allele. Nonetheless, the percentage of fast acetylators is also notably high, accounting for 40% of this population [15,16].

Based on the results obtained in the present study, the observed frequency of the SNP c.857G>A was 8.5% (mutant heterozygote/c. 857GA), similar to the frequency of 9.6% identified in a study conducted in Brazil [23]. After describing these frequencies and continuing the statistical analyses, a *p* value <0.05 was observed in the association between the presence of this variant and the treatment duration. The SNP c.857G>A appears to confer protection, indicating a higher efficacy of anti-TB treatment within six months for carriers of this allele.

The substitution of a glycine amino acid for a glutamate at position 286 of the protein results in a change in the metabolization capacity, making it slower and leading to the drug being retained in the body for longer period. While this may increase the probability of hepatotoxicity, it is more likely that the drug will reach optimal levels necessary for a favorable therapeutic response.

In Brazil, the presence of a diverse array of SNPs is a result of the extensive miscegenation of the population [15]. In this study, we found a predominance of slow acetylators (47.9%), similar to the proportions observed in the African population (46%) and the European population (58%) [16]. The most prevalent allele was NAT2*5B, accounting for 38.2% of the cases.

Slow acetylators tend to have a higher risk of developing drug-induced hepatitis due to their significantly lower INH clearance rate, which is approximately three times slower compared to that of fast acetylators. On the other hand, fast acetylators are susceptible to a drop in serum drug concentration because of their high rate of clearance and excretion. This may contribute to a delayed therapeutic response or even treatment failure [16,17].

Studies in silico highlight that the fast acetylation phenotype represents a significant risk factor in the development of resistance to INH. By developing a three-dimensional mutant model of human N-acetyltransferase 2 from the wild type, the interaction of these proteins with acetyl-INH was simulated, demonstrating a higher affinity of the mutant model for this molecule. Thus, the increase in the acetylation rate resulted in an intensification in the formation of acetyl-INH through the inactivation of INH, leading to minimal exposure of INH to MTB and consequently fostering the emergence of resistance [13,16,24]. 

In a prior study conducted by our research group, Teixeira et al. [22] identified the three most frequent *NAT2* alleles in the city of Rio de Janeiro, and their frequencies align with the findings of the present study. Among these alleles, one leads to the fast acetylation phenotype (NAT2*4), occurring at a frequency of 20%, and two alleles, NAT2*5B and NAT2*6A, resulted in a slow acetylation phenotype and had frequencies of 33 and 26%, respectively.

The alleles NAT2*new5 (0.5%) and NAT2*new6 (1%) were named as such because they present the SNP common to clusters *5 (c.341T>C) and *6 (c.590G>A). However, the specific combination of these with other SNPs identified in the same sequence have not been characterized in previous studies. Consequently, their impact on the acetylation phenotype is unknown.

Given the diversity of polymorphisms in the NAT2 gene across different populations, it is essential to genotype patients with TB before prescribing INH. This approach allows for dose adjustments and enhances treatment efficacy, leading to improved patient quality of life as well as potential reductions in the costs associated with necessary treatment in cases of unfavorable outcomes.

## 5. Conclusions

The presence of the SNP c.857G>A was statistically more prevalent in the group of patients who were cured of TB within 6 months, suggesting that it serves as a genetic marker for protection against delayed treatment time. This is attributed to its association with a slow acetylation phenotype, which allows for the maintenance of adequate serum levels of INH in the body.

A statistically significant relationship was established between the occurrence of complications (positive BAAR/culture and worsening/no improvement of radiographic images after 2 months of treatment) and the necessity of extending treatment durations.

Different NAT2 genotypes primarily influence the rate at which INH is cleared from the body and, consequently, its serum concentration. Thus, if these genetic variations are considered in the context of medical practice and health policy in Brazil, several implications could arise: (a) Personalized Medicine—This would be ensured by genotyping patients for *NAT2* variants before prescribing INH. This means tailoring the dosage of INH based on an individual’s genetic makeup, optimizing treatment effectiveness and potentially reducing adverse effects. (b) Improved Treatment Outcomes. These would be attained by adjusting the INH dosage based on NAT2 genotypes. This personalized approach could result in better patient responses to therapy and potentially reduce the risk of treatment failure or relapse. (c) Cost-Efficacy: Although genotyping involves additional costs, the potential benefits in terms of treatment efficacy could lead to cost savings in the long run. Preventing treatment failures or relapses can reduce the economic burden associated with extended or repeat treatments, hospitalizations, and additional healthcare services. (d) Public Health Impact: Implementing genotyping in TB treatment aligns with the broader goal of precision medicine and could contribute to improved public health outcomes. If Brazil adopts a genotyping approach, it may set a precedent for other countries facing similar challenges in TB treatment and could influence healthcare policies related to TB treatment in Brazil. Policies may be updated to include guidelines on genotyping before prescribing INH, and healthcare providers may be encouraged or mandated to incorporate genetic testing into their TB management protocols.

## Figures and Tables

**Table 1:** Frequency of SNPs identified in the *NAT2* gene in individuals with and without pulmonary TB.

*NAT2*	**Genotype Frequency**			
	**SNP**		**Pulmonary TB (%) *n* = 136**	**Non-TB Carriers (%)** ***n* = 168**
	c.191G>A	GG	129 ^(94.8)^	156 ^(92.8)^
		GA	4 ^(2.9)^	3 ^(1.7)^
		AA	-	-
	c.247G>A	GG	132 ^(97)^	159 ^(94.6)^
		GA	1 ^(0.7)^	-
		AA	-	-
	c.282C>T	CC	53 ^(38.9)^	75 ^(44.6)^
		CT	67 ^(49.2)^	64 ^(38)^
		TT	13 ^(9.5)^	20 ^(11.9)^
	c.341T>C	TT	53 ^(38.9)^	64 ^(38)^
		TC	58 ^(42.6)^	71 ^(42.2)^
		CC	22 ^(16.1)^	24 ^(14.2)^
	c.481C>T	CC	58 ^(42.6)^	70 ^(41.6)^
		CT	59 ^(43.3)^	71 ^(42.2)^
		TT	16 ^(11.7)^	18 ^(10.7)^
	c.578C>T	CC	132 ^(97)^	158 ^(94)^
		CT	1 ^(7.3)^	1 ^(0.5)^
		TT	-	-
	c.590G>A	GG	73 ^(53.6)^	93 ^(55.3)^
		GA	48 ^(35.2)^	53 ^(31.5)^
		AA	12 ^(8.8)^	13 ^(7.7)^
	c.803A>G	AA	56 ^(41.1)^	63 ^(37.5)^
		AG	50 ^(36.7)^	66 ^(39.2)^
		GG	27 ^(19.8)^	30 ^(17.8)^
	c.857G>A	GG	119 ^(87.5)^	148 ^(88)^
		GA	14 ^(10.2)^	11 ^(6.5)^
		AA	-	-
	ND *		3	9

* ND = Not defined (cases in which it was not possible to perform an analysis of the obtained sequences).

**Table 2:** *NAT2* genotype/phenotype.

	Haplotype ^2^	Phenotype	Non-TB Carriers (%) *n* = 168	Pulmonary TB (%) *n* = 136
NAT2*4 ^1^	GGGCTCCCACCGAGTCAAGG	Rapid	75 ^(23.5)^	40 ^(20.3)^
NAT2*12A	GGGCTCCCACCGAGTCA**G**GG	Rapid	11 ^(3.4)^	9 ^(4.6)^
NAT2*12B	GGG**T**TCCCACCGAGTCA**G**GG	Rapid	2 ^(0.6)^	-
NAT2*13A	GGG**T**TCCCACCGAGTCAAGG	Rapid	14 ^(4.4)^	6 ^(3.0)^
NAT2*12C	GGGCTCCCA**T**CGAGTCA**G**GG	Rapid	2 ^(0.6)^	3 ^(1.5)^
NAT2*5A	GGGC**C**CCCA**T**CGAGTCAAGG	Slow	8 ^(2.5)^	5 ^(2.5)^
NAT2*5B	GGGC**C**CCCA**T**CGAGTCA**G**GG	Slow	99 ^(31.1)^	62 ^(31.5)^
NAT2*5C	GGGC**C**CCCACCGAGTCA**G**GG	Slow	12 ^(3.7)^	6 ^(3.0)^
NAT2*5D	GGGC**C**CCCACCGAGTCAAGG	Slow	2 ^(0.6)^	1 ^(0.5)^
NAT2*6A	GGG**T**TCCCACC**A**AGTCAAGG	Slow	73 ^(22.9)^	45 ^(22.9)^
NAT2*6B	GGGCTCCCACC**A**AGTCAAGG	Slow	4 ^(1.2)^	3 ^(1.5)^
NAT2*7B	GGG**T**TCCCACCGAGTCAAG**A**	Slow	11 ^(3.4)^	8 ^(4.0)^
NAT2*14B	**A**GG**T**TCCCACCGAGTCAAGG	Slow	3 ^(0.9)^	4 ^(2.0)^
NAT2*5J	GGG**TC**CCCACC**A**AGTCAAGG	Slow	-	1 ^(0.5)^
NAT2*6F	GGGCTCCCACC**A**AGTCA**G**GG	ND ^3^	1 ^(0.3)^	-
NAT2*12E	GGG**T**TCCCAC**T**GAGTCA**G**GG	ND	1 ^(0.3)^	-
NAT2*5KA	GGG**TC**CCCACCGAGTCAAG**A**	ND	-	1 ^(0.5)^
NAT2*new5	GGGC**C**CCCA**T**C**A**AGTCAAGG	ND	-	1 ^(0.5)^
NAT2*new6	GG**AT**TCCCACC**A**AGTCAAGG	ND	-	2 ^(1.0)^
Total			318	197
ND			18	76

^1^ Reference allele (without the presence of variants); ^2^ Nucleotide positions in the *NAT2* coding region and their respective changes (SNPs): c.191G>A, c.247G>A, c.282 C>T, c.341 T>C, c.481C>T, c.578C>T, c.590G>A, c.803A>G, c.857G>A; ^3^ ND = Not determined.

**Table 3:** Distribution of the *NAT2* genotypes and phenotypes among TB and non-TB patients.

	Pulmonary TB	No TB	Total
Fast acetylators	15 ^(11.3)^	18 ^(11.3)^	33 ^(11.3)^
NAT2*4/*4	8	9	17 ^(5.8)^
NAT2*4/*12A	1	1	2 ^(0.7)^
NAT2*4/*13A	4	6	10 ^(3.4)^
NAT2*12C/*12C	1	1	2 ^(0.7)^
NAT2*12B/*12B	-	1	1 ^(0.3)^
NAT2*12A/*13A	1	-	1 ^(0.3)^
Intermediate acetylators	45 ^(33.8)^	68 ^(42.8)^	113 ^(38.7)^
NAT2*4/*5A	3	4	7 ^(2.4)^
NAT2*4/*5B	7	24	31 ^(10.6)^
NAT2*4/*5C	2	2	4 ^(1.4)^
NAT2*4/*5D	1	-	1 ^(0.3)^
NAT2*4/*6A	11	16	27 ^(9.2)^
NAT2*4/*6B	2	-	2 ^(0.7)^
NAT2*4/*7B	4	3	7 ^(2.4)^
NAT2*4/*14B	1	1	2 ^(0.7)^
NAT2*5B/*13A	2	4	6 ^(2.1)^
NAT2*12A/*5B	5	5	10 ^(3.4)^
NAT2*12A/*5C	1	-	1 ^(0.3)^
NAT2*12A/*6A	3	3	6 ^(2.1)^
NAT2*12A/*6B	-	1	1 ^(0.3)^
NAT2*12A/*7B	-	1	1 ^(0.3)^
NAT2*12C/*6A	1	-	1 ^(0.3)^
NAT2*13A/*6A	2	4	6 ^(2.1)^
Slow acetylators	69 ^(51.9)^	71 ^(44.6)^	140 ^(47.9)^
NAT2*5A/*14B	1 ^(0.75)^	-	1 ^(0.3)^
NAT2*5A/*6A	3	3	6 ^(2.1)^
NAT2*5B/*14B	2 ^(1.5)^	2	4 ^(1.4)^
NAT2*5B/*5B	15	17	32 ^(11.0)^
NAT2*5B/*6A	21	19	40 ^(13.7)^
NAT2*5B/*7B	7	4	11 ^(3.8)^
NAT2*5C/*5B	5	3	8 ^(2.7)^
NAT2*5C/*5C	-	2	2 ^(0.7)^
NAT2*5C/*5J	1 ^(0.75)^	-	1 ^(0.3)^
NAT2*5C/*6A	-	1	1 ^(0.3)^
NAT2*5C/*7B	1 ^(0.75)^	1	2 ^(0.7)^
NAT2*5D/*5A	-	1	1 ^(0.3)^
NAT2*5D/*5C	-	1	1 ^(0.3)^
NAT2*6A/*6A	8	12	20 ^(6.8)^
NAT2*6B/*5B	1 ^(0.75)^	2	3 ^(1.0)^
NAT2*6B/*6A	2 ^(1.5)^	-	2 ^(0.7)^
NAT*6B/*6B	-	1	1 ^(0.3)^
NAT2*7B/*6A	2 ^(1.5)^	2	4 ^(1.4)^
Undetermined acetylation	4 ^(3)^	2 ^(1.3)^	6 ^(2)^
NAT2*new5/*6A	1 ^(0.75)^	-	1 ^(0.3)^
NAT2*12A/*new6	1 ^(0.75)^	-	1 ^(0.3)^
NAT2*6A/*new6	1 ^(0.75)^	-	1 ^(0.3)^
NAT2*6A/*12E	-	1	1 ^(0.3)^
NAT2*6F/*5B	-	1	1 ^(0.3)^
NAT2*5D/*5KA	1 ^(0.75)^	-	1 ^(0.3)^
Total	133 ^(100)^	159 ^(100)^	292 ^(100)^
Not determined	3	9	12
Total	136	168	304

**Table 4:** Association between mutant alleles in candidate genes and unfavorable outcomes in the 2nd month of treatment for pulmonary TB.

	SNPs	Presence of at Least One Mutant Allele	Unfavorable Outcome in the 2nd Month			
			Absence ^(%)^	Presence ^(%)^	Q (*p*) ^1^	OR ^2^
*NAT2*	191A	no	70 ^(74.5)^	24 ^(25.5)^	0.001 (0.981)	0.972 IC (0.096–9.796)
		yes	3 ^(75.0)^	1 ^(25.0)^		
	247A	no	72 ^(74.2)^	25 ^(25.8)^	0.346 (0.556)	0.742 IC (0.660–0.835)
		yes	1 ^(100.0)^	0 ^(0)^		
	282T	no	31 ^(73.8)^	11 ^(26.2)^	0.018 (0.894)	0.939 IC (0.376–2.348)
		yes	42 ^(75.0)^	14 ^(25.0)^		
	341C	no	30 ^(75.0)^	10 ^(25.0)^	0.009 (0.923)	1.047 IC (0.415–2.642)
		yes	43 ^(74.1)^	15 ^(25.9)^		
	481T	no	31 ^(75.6)^	10 ^(24.4)^	0.047 (0.829)	1.107 IC (0.439–2.792)
		yes	42 ^(73.7)^	15 ^(26.3)^		
	578T	no	72 ^(74.2)^	25 ^(25.8)^	0.346 (0.556)	0.742 IC (0.660–0.835)
		yes	1 ^(100.0)^	0 ^(0.0)^		
	590A	no	41 ^(73.2)^	15 ^(26.8)^	0.112 (0.738)	0.854 IC (0.339–2.152)
		yes	32 ^(76.2)^	10 ^(23.8)^		
	803G	no	30 ^(75.0)^	10 ^(25.0)^	0.009 (0.923)	1.047 IC (0.415–2.642)
		yes	43 ^(74.1)^	15 ^(25.9)^		
	857A	no	66 ^(74.2)^	23 ^(25.8)^	0.056 (0.812)	0.820 IC (0.159–4.233)
		yes	7 ^(77.8)^	2 ^(22.2)^		

^1^ Chi-square (*p* value); ^2^ odds ratio.

**Table 5:** Presence of at least one mutant allele in candidate genes and its prevalence in individuals with TB according to the treatment duration.

		Treatment Period < 9 Months		Treatment Period > 9 Months				
**Presence of at least one mutant allele of *NAT2***	**SNPs**	**Absence ^(%)^**	**Presence** **^(%)^**	**Absence** **^(%)^**	**Presence** **^(%)^**	* Mann-Whitney Test	** Amino Acid Change	**Reference Sequence (NCBI)**
	191A	62 ^(98.4)^	1 ^(1.6)^	32 ^(91.4)^	3 ^(8.6)^	-	Arg64Gln	rs1801279
	247A	62 ^(98.4)^	1 ^(1.6)^	35 ^(100)^	-	0.918	Gly83Ser	rs746734312
	282T	24 ^(38.1)^	39 ^(61.9)^	18 ^(51.4)^	17 ^(48.6)^	0.063	Tyr94Tyr	rs1041983
	341C	26 ^(41.3)^	37 ^(58.7)^	14 ^(40.0)^	21 ^(60.0)^	0.985	Ile114Thr	rs1801280
	481T	28 ^(44.4)^	35 ^(55.6)^	13 ^(37.1)^	22 ^(62.9)^	0.656	Leu161Leu	rs1799929
	578T	62 ^(98.4)^	1 ^(1.6)^	35 ^(100)^	-	0.469	Thr193Met	rs79050330
	590A	34 ^(54.0)^	29 ^(46.0)^	22 ^(62.9)^	13 ^(37.1)^	0.273	Arg197Gln	rs1799930
	803G	26 ^(41.3)^	37 ^(58.7)^	14 ^(40.0)^	21 ^(60.0)^	0.525	Lys268Arg	rs1208
	857A	54 ^(85.7)^	9 ^(14.3)^	35 ^(100)^	-	0.008	Gly286Glu	rs1799931
	Total	63		35				

* *p* value; ** Arg = Arginine; Gln = Glutamine; Gly = Glycine; Ser = Serine; Tyr = Tyrosine; Thr = Threonine; Leu = Leucine; Met = Methionine; Lys = Lysine; Glu = Glutamate; Asn = Asparagine; Asp = Aspartate; Ser = Serine; Pro = Proline; Ala = Alanine; Ile = Isoleucine.

## Data Availability

All data generated or analyzed in this study are available from the corresponding author on reasonable request.

## References

[1] WHO (2022). Global Tuberculosis Report 2022.

[2] (2022). Epidemiological Tuberculosis Bulletin.

[3] WHO (2021). Global Tuberculosis Report 2021.

[4] Information System for Notifiable Diseases Tuberculosis. http://sistemas.saude.rj.gov.br/tabnetbd/webtabx.exe?sinan/tf_tuberculose.def/.

[5] Mah A., Kharrat H., Ahmed R., Gao Z., Der E., Hansen E., Long R., Kunimoto D., Cooper R. (2015). Serum drug concentrations of INH and RMP predict 2-month sputum culture results in tuberculosis patients. Int. J. Tuberc. Lung Dis..

[6] Yee S.W., Brackman D.J., Ennis E.A., Sugiyama Y., Kamdem L.K., Blanchard R., Galetin A., Zhang L., Giacomini K.M. (2018). Influence of Transporter Polymorphisms on Drug Disposition and Response: A Perspective from the International Transporter Consortium. Clin. Pharmacol. Ther..

[7] Ingelman-Sundberg M. (2001). Pharmacogenetics: An opportunity for a safer and more efficient pharmacotherapy. J. Intern. Med..

[8] Evans D.A.P. (1989). N-acetyltransferase. Pharmacol. Ther..

[9] Wu H., Dombrovsky L., Tempel W., Martin F., Loppnau P., Goodfellow G.H., Grant D.M., Plotnikov A.N. (2007). Structural basis of substrate-binding specificity of human arylamine N-acetyltransferases. J. Biol. Chem..

[10] Hickman D., Risch A., Buckle V., Spurr N.K., Jeremiah S.J., McCarthy A., Sim E. (1994). Chromosomal localization of human genes for arylamine *N*-acetyltransferase. Biochem. J..

[11] Hein D.W., Doll M.A., Fretland A.J., Leff M.A., Webb S.J., Xiao G.H., Devanaboyina U.S., Nangju N.A., Feng Y. (2000). Molecular genetics and epidemiology of the NAT1 and NAT2 acetylation polymorphisms. Cancer Epidemiol. Biomark. Prev..

[12] Teixeira R.L.D.F., Morato R.G., Cabello P.H., Muniz L.M.K., Moreira A.D.S.R., Kritski A.L., Santos A.R. (2011). Genetic polymorphisms of NAT2, CYP2E1 and GST enzymes and the occurrence of antituberculosis drug-induced hepatitis in Brazilian TB patients. Memórias Do Inst. Oswaldo Cruz.

[13] Azuma J., Ohno M., Kubota R., Yokota S., Nagai T., Tsuyuguchi K., Okuda Y., Takashima T., Kamimura S., Pharmacogenetics-Based Tuberculosis Therapy Research Group (2012). NAT2 genotype guided regimen reduces isoniazid-induced liver injury and early treatment failure in the 6-month four-drug standard treatment of tuberculosis: A randomized controlled trial for pharmacogenetics-based therapy. Eur. J. Clin. Pharmacol..

[14] Donald P.R., Sirgel F.A., Venter A., Parkin D.P., Seifart H.I., van de Wal B.W., Werely C., van Helden P.D., Maritz J.S. (2004). The Influence of Human N-Acetyltransferase Genotype on the Early Bactericidal Activity of Isoniazid. Clin. Infect. Dis..

[15] Choi R., Jeong B.-H., Koh W.-J., Lee S.-Y. (2017). Recommendations for Optimizing Tuberculosis Treatment: Therapeutic Drug Monitoring, Pharmacogenetics, and Nutritional Status Considerations. Ann. Lab. Med..

[16] Jing W., Zong Z., Tang B., Wang J., Zhang T., Wen S., Xue Y., Chu N., Zhao W., Huang H. (2020). Population Pharmacokinetic Analysis of Isoniazid among Pulmonary Tuberculosis Patients from China. Antimicrob. Agents Chemother..

[17] Jung J.A., Lee S.-Y., Kim T.-E., Kwon O.J., Jeon K., Jeong B.-H., Park H.Y., Ko J.-W., Choi R., Woo H.-I. (2015). A proposal for an individualized pharmacogenetic-guided isoniazid dosage regimen for patients with tuberculosis. Drug Des. Dev. Ther..

[18] Lopes M.Q.P., Teixeira R.L.F., Cabello P.H., Nery J.A.C., Sales A.M., JR E.P.N., Moreira M.V., Stahlke E.V.R., Possuelo L.G., Rossetti M.L.R. (2023). Human N-acetyltransferase 2 (NAT2) gene variability in Brazilian populations from different geographical areas. Front. Pharmacol..

[19] Stephens M., Donnelly P. (2003). A comparison of bayesian methods for haplotype reconstruction from population genotype data. Am. J. Hum. Genet..

[20] Fretland A.J., Leff M.A., Doll M.A., Hein D.W. (2001). Functional characterization of human N-acetyltransferase 2 (NAT2) single nucleotide polymorphisms. Pharmacogenetics.

[21] Dandara C., Masimirembwa C.M., Magimba A., Kaaya S., Sayi J., Sommers D.K., Snyman J.R., Hasler J.A. (2003). Arylamine N-acetyltransferase (NAT2) genotypes in Africans: The identification of a new allele with nucleotide changes 481C:>: T and 590G:>: A. Pharmacogenet. Genom..

[22] Teixeira R.L., Miranda A.B., Pacheco A.G., Lopes M.Q., Fonseca-Costa J., Rabahi M.F., Melo H.M., Kritski A.L., Mello F.C., Suffys P.N. (2007). Genetic profile of the arylamine N-acetyltransferase 2 coding gene among individuals from two different regions of Brazil. Mutat. Res. Mol. Mech. Mutagen..

[23] Heinrich M.M., Zembrzuski V.M., Ota M.M., Sacchi F.P., Teixeira R.L., Acero P.H.C., Cunha G.M., Souza-Santos R., Croda J., Basta P.C. (2016). Factors associated with anti-TB drug-induced hepatotoxicity and genetic polymorphisms in indigenous and non-indigenous populations in Brazil. Tuberculosis.

[24] Unissa A.N., Sukumar S., Hanna L.E. (2017). The role of N-acetyl transferases on isoniazid resistance from Mycobacterium tuberculosis and human: An in silico approach. Tuberc. Respir. Dis..

